# Keampferol-3-O-rhamnoside abrogates amyloid beta toxicity by modulating monomers and remodeling oligomers and fibrils to non-toxic aggregates

**DOI:** 10.1186/1423-0127-19-104

**Published:** 2012-12-21

**Authors:** Md Golam Sharoar, Arjun Thapa, Mohammad Shahnawaz, Vijay Sankar Ramasamy, Eun-Rhan Woo, Song Yub Shin, Il-Seon Park

**Affiliations:** 1 Department of Bio-materials Engineering, Chosun University, Gwanju, 501-759, Republic of Korea; 2 Department of Cellular and Molecular Medicine, Chosun University, Gwanju, 501-759, Republic of Korea; 3 College of Pharmacy, Chosun University, Gwanju, 501-759, Republic of Korea; 4 Department of Genetic Engineering and Biotechnology, University of Rajshahi, Rajshahi, 6205, Bangladesh; 5 Department of Cell Biology, University of Oklahoma Health Sciences Center, Oklahoma City, OK, USA; 6 Medical School, The University of Texas Health Science Center at Houston, 6431 Fannin St, Houston, TX, 77030, USA

**Keywords:** Aβ, Kaempferol-3-O-rhamnoside, Oligomer, Aggregation, Cytotoxicity, Alzheimer’s disease

## Abstract

**Background:**

Aggregation of soluble, monomeric β- amyloid (Aβ) to oligomeric and then insoluble fibrillar Aβ is a key pathogenic feature in development of Alzheimer’s disease (AD). Increasing evidence suggests that toxicity is linked to diffusible Aβ oligomers, rather than to insoluble fibrils. The use of naturally occurring small molecules for inhibition of Aβ aggregation has recently attracted significant interest for development of effective therapeutic strategies against the disease. A natural polyphenolic flavone, Kaempferol-3-O-rhamnoside (K-3-rh), was utilized to investigate its effects on aggregation and cytotoxic effects of Aβ42 peptide. Several biochemical techniques were used to determine the conformational changes and cytotoxic effect of the peptide in the presence and absence of K-3-rh.

**Results:**

K-3-rh showed a dose-dependent effect against Aβ42 mediated cytotoxicity. Anti-amyloidogenic properties of K-3-rh were found to be efficient in inhibiting fibrilogenesis and secondary structural transformation of the peptide. The consequence of these inhibitions was the accumulation of oligomeric structural species. The accumulated aggregates were smaller, soluble, non-β-sheet and non-toxic aggregates, compared to preformed toxic Aβ oligomers. K-3-rh was also found to have the remodeling properties of preformed soluble oligomers and fibrils. Both of these conformers were found to remodel into non-toxic aggregates. The results showed that K-3-rh interacts with different Aβ conformers, which affects fibril formation, oligomeric maturation and fibrillar stabilization.

**Conclusion:**

K-3-rh is an efficient molecule to hinder the self assembly and to abrogate the cytotoxic effects of Aβ42 peptide. Hence, K-3-rh and small molecules with similar structure might be considered for therapeutic development against AD.

## Background

Alzheimer’s disease (AD) is characterized by progressive and insidious neurodegeneration of the central nervous system, which eventually leads to a gradual decline of cognitive function and dementia [1]. The principal neuropathological features of AD are the presence of intracellular neurofibrillary tangles and extracellular deposition of amyloid beta (Aβ) peptides in the form of senile plaques [2]. The peptide is derived from amyloid precursor protein (APP) by sequential cleavage of α-, β-, and γ- secretases [3]. Differential cleavage by γ-secretase is one factor contributing to formation of Aβ with different C-termini. Among the two most common alloforms of Aβ, Aβ40 (ending at Val at 40 position) and Aβ42 (ending at Ala at 42 position), the longer form(Aβ42) is more prone to aggregation than the shorter form(Aβ40), and is the main constituent of senile plaques [4].

The native Aβ peptides spontaneously undergo conformational changes to form β-sheets rich insoluble fibril through fibrillization process. Fibrillization follows nucleation-dependent aggregation, which basically comprises two phases; nucleation, the formation of the nucleus by association of a series of monomeric peptides [5], and extension, the subsequent addition of monomers to the end of the existing nucleus [6]. However, Aβ fibrillization derives several transitional species including trimer, pentamer, or higher molecular weight complex, also known as Aβ -derived diffusible ligands (ADDLs) [7], oligomers composed of 15–20 monomers [8], protofibrils (string of oligomers) [9], and dodecameric oligomers Aβ *56 [10]. All of these unstable intermediates are collectively designated as “soluble Aβ” [11]. Recent studies have reported that the soluble Aβ oligomers formed during early aggregation are the main cytotoxic agents rather than monomeric or fibrilar forms [12]. Therefore, preventing the assembly of Aβ monomer into toxic oligomer or fibril is the primary goal of a number of therapeutic strategies under development or in clinical trials. Hence, major research has involved the development of compounds capable of inhibiting or reversing the Aβ aggregation process. Thus far, a number of diverse compounds including small molecules [13,14], antibodies [15], peptidic β-sheet breakers [16,17], and osmolytes [18], have been used to prevent or to reduce the aggregation of Aβ into oligomers or fibrils. Small molecules have been reported to inhibit single or multiple steps of the Aβ fibrillization process [19]. In addition, small molecules that efficiently inhibit the early steps of Aβ fibrillogenesis and stabilize its nontoxic conformations have been considered as possible AD therapeutics, because the self-assembly of the peptide is directly linked to the pathogenesis of AD. Among the Aβ interacting small molecules, curcumin is one of the most attractive compounds to scientists due to its direct capability to bind with small Aβ species, to block aggregation and fibril formation and to disaggregate mature fibrils under both *in vitro* and *in vivo* conditions [20]. Other Aβ binding molecules includes sulfonate dye Congo red [21] and thioflavin T(ThT) [22], which are utilized as classic reagents for determination of characteristic β-sheet mediated fibrillization. In fact, the most of reported small molecule Aβ inhibitors are structurally similar to Congo red and ThT, in that they are planar and aromatic compounds.

Flavonoids, found ubiquitously in plants, are the most common polyphenolic compounds group in human diet form [23]. This group of compounds has several beneficial effects on human health such as anti-oxidant [24], anti-allergic, anti-cancer and anti-inflammatory [25], and anti-microbial [26] activities. Flavonoids are also reported to decrease the risk of age related dementia [27]. Extensive studies on *Ginkgo biloba* extracts HE208 [28] and EGb 761 [29] indicate that the flavonoid molecules are essential for anti-amyloidogenic and anti-apoptotic activity in neural cells. A number of isolated flavonoids have been found to be effective against oligomer formation, fibril stabilization and cytotoxic effects of Aβ peptide [30-34]. On the other hand, some polyphenoles are reported to be inhibited Aβ fibrilogenesis, but not Aβ mediated cytotoxicity, while others described as cytoprotective, but not antifibrillogenic against Aβ [35,36]. Hence, the correlation of anti-amyloidogenic activity and anti-cytotoxic effect of flavonoids remains unclear. In the current study, we screened several phenolic compounds against cytotoxic effects of Aβ peptide. We identified a polyphenolic glycoside flavone, Keampferol-3-rhamnoside (K-3-rh), as an effective molecule for alteration of the on pathway aggregation of different Aβ conformers to off pathway non-toxic species, as well as for disaggregation preformed mature fibrils.

## Methods

### Materials

K-3-rh, quercitrin (Q) and keampferol-3-rutinoside (K-3-ru) were purchased from Sigma (St. Louis, USA). Gallic acid (GA), protocatechuic acid (PA), gallic acid methyl ester (GAME), quercetin dihydrate (Quer-di-hy), quercetin hydrate (Quer-hy) and kaempferol (K) are isolated as described earlier [37]. Fetal Bovine Serum (FBS) was purchased from Life Technology Inc. (Grand Island, USA). Dulbecco’s modified Eagles medium, Ham’s F 12 (1:1) (DMEM/F-12) was obtained from Welgene (Daegu, Korea). Western blotting detection kit (WEST-ZOL plus) was purchased from iNtron Biotechnology (Gyeonggi-do, Korea). Phosphate buffered saline (PBS) was purchased from Amresco (Solon, USA). Monoclonal anti-Aβ antibody 6E10 was acquired from Signet Laboratories (Dedham, USA). Urea was obtained from USB chemicals and acetonitrile was from Merck (Darmstadt, Germany). All other chemicals were obtained from Sigma (St. Louis, USA), unless otherwise stated.

### Preparation of amyloid beta

Amyloid beta peptides were expressed in *E.coli* as fusion proteins and purified as described before [38]. The purified peptides were solubilized in 100% 1,1,1,3,3,3,-hexafluoro-2-propanol, and dried under nitrogen flow and subsequently, under a vacuum for 30 min. The peptide aliquots were stored at −20°C until use. Immediately before use, the peptides were dissolved in 0.1% NH_4_OH at a concentration of 2 mg/ml followed by bath sonication for 10 min at 4°C. The solution was diluted at the desired concentration with PBS. Aβ42 oligomers and fibrils were prepared as described earlier [39] with little modification. Briefly, oligomers were prepared by diluting the peptides in cell culture media at a concentration of 100 μM, vortexing for 30 seconds and incubating at 4°C for 12 h. K-3-rh accumulated oligomeric species were prepared by co-incubating Aβ42 (20 μM) and 40 μM K-3-rh for 12 h and centrifuging the sample to obtain the supernatant. To make fibrils, Aβ42 (100 μM) was incubated in the presence of 0.02% sodium azide in PBS at 37°C for four days. The samples were then centrifuged at 16000 × g for 30 min. The pellet fraction (fibrils) was washed three times with PBS. Fibrils were sonicated for 10 min, quantified using the Bradford method and used immediately or stored at −80°C.

### Cell culture and cell death assay

Human neuroblastoma SH-SY5Y was cultured in Dulbecco’s Modified Eagles medium and Ham’s F 12 (1:1), supplemented with 10% (v/v) fetal bovine serum (FBS) and 1% antibiotics, at 37°C under 5% CO_2_. Cells were seeded at a density of 15,000 cells/well in 96-well plates (Nunc, Denmark) and incubated for 24 h. The media were replaced with serum-free media and cells were further cultured for 24 h. For measurement of cell death, cells were treated with indicated concentrations of different Aβ conformers in the presence or absence of flavonoid(s) for 12 or 24 h. Cell viability was assessed by MTT reduction assay. Briefly, 20 μl of 5 mg/ml MTT solution in PBS was added to each well and incubated for 2 h. Then, 100 μl of solubilization buffer [20% SDS solution in 50% (v/v) DMF (pH 4.7)] was added to each well. The absorbance was recorded after 12–16 h at 570 nm using a micro plate reader Spectra Max 190 (Molecular Devices, CA, USA).

### Thioflavin-T (Th-T) assays

For the polymerization assay, Aβ42 (20 μM) was incubated in PBS at 37°C in the presence or absence of K-3-rh in a final volume of 30 μl without shaking. Twenty μl from each reaction was mixed with 80 μl of 5 μM Th-T in PBS solution. Fluorescence was measured on a microplate spectrofluorometer Gemini-XS (Molecular Devices CA, USA) using excitation at 440 nm and emission at 490 nm [39]. Th-T fluorescence representing the characteristic sigmoidal curve was plotted as common logarithms in the equation: log *F* (*t*)/ *A−F* (*t*)] = *at* + *b*, where *t* is the reaction time, *F*(*t*) is the fluorescence as a function of time, *A* is tentatively determined as *F* (∞), *a* slope and *b* is the *y-* intercept [40]. Differentiating the above equation by *t* and subsequent rearrangement produced a logistic equation, *F’* (*t*) = *BF* (*t*) *A*−*F* (*t*)], where, *B* = *a* ln 10 *A* / *2*, and *F’*(*t*) represents rate of fluorescence increase at a given time. When *F*(*t*) = *A*/*2*, *F*(*t*)/ *A−F*(*t*) = 1 and *F’*(*t*) reaches its maximum. This time point was referred to as *t* / *2*[40]. For the Aβ42 fiber extension assay, fresh Aβ42 (20 μM) was incubated with preformed Aβ42 fibrils (1.1 μM) in the presence of varying concentrations of K-3-rh. For the fibril destabilization assay, preformed Aβ42 fibrils (20 μM) were incubated in presence of varying concentrations of K-3-rh.

### Circular dichroism (CD) spectroscopy

Aβ42 (20 μM) was incubated in PBS at 37°C either alone or in the presence of 20 μM of K-3-rh for 0 or 12 h. CD spectra [41] were recorded using a 1-mm path length cuvette at 0.5 nm intervals between 190 nm and 250 nm at 1 nm resolution with a scan rate of 50 nm/min using a Jasco Sectropolarimeter (Jasco Co., Tokyo, Japan) at 25°C. Average was taken from five scans for each sample. Aβ spectra were obtained by subtracting buffer background. Background spectra given by K-3-rh alone under identical conditions were subtracted from Aβ samples incubated in the presence of K-3-rh. The corrected, averaged spectra were smoothed using the means-movement algorithm in the Jasco spectra analysis program.

### Transmission Electron Microscopy (TEM)

Twenty μM native Aβ42, oligomeric Aβ42(OAβ42) or fibrilar Aβ42(fAβ42) either alone or in the presence of K-3-rh was incubated in PBS at 37°C for 12 h. Five μl of sample was adsorbed on Formvar-coated 200-mesh nickel grids for 30 min and extra solution was wiped off [42]. The grids were negatively stained with 2% uranyl acetate for 1 min and washed at least three times with distilled water. The samples were then analyzed using transmission electron microscopy (Hitachi, Japan) at an accelerating voltage of 80 kV at magnification of 40,000x.

### Detection of Aβ structural species by immunoblotting

Twenty μM native Aβ42 was incubated in PBS at 37°C without or with indicated amounts of K-3-rh for 12 and 24 h. 2 μl from each reaction mixture was diluted with 8 μl or 6 μl (for cross link reaction) of PBS. For control (0 h) fresh Aβ42 was diluted to a concentration of 20 μM in PBS and from there 2 μl was taken. 2 μl of SDS buffer [50 mM Tris buffer (pH 6.8), 10% glycerol, 2% SDS, and 0.1% β-mercaptoethanol] was then added to each sample. Crosslinking of the peptide in the reaction mixture was performed as described previously [43], with slight modification. Briefly, before addition of SDS buffer, each sample was incubated with 0.01% glutaraldehyde (v/v) in PBS (2 μl from a 0.05% stock solution) for 10 min. The cross-linking reaction was then terminated with an equal volume of SDS buffer. Samples were then run on a SDS-PAGE gel (16% acrylamide) without boiling. Subsequently, the peptide was transferred to polyvinylidene difluoride (PVDF) membrane. After blocking with 5% milk in Tris-buffered saline containing 0.2% (v/v) Tween 20 at room temperature for 1 h, the membranes were probed with anti-Aβ antibody 6E10 (1:10,000). The blots were then incubated with horseradish peroxidase-conjugated Ig anti-mouse antibody (1:5,000) for 1 h at room temperature and developed using a West-zol plus kit.

### Statistical analysis

All experiments were performed in triplicate. For each experiment, data are expressed as the mean ± standard error (SE, n = 3) and statistical significance was assessed by one-way analysis of variance (ANOVA) and Student’s t-tests. A *p* value of <0.05 was considered significant.

## Results

### K-3-rh and related flavonoids are cytoprotective against Aβ42 toxicity

**Figure 1:**
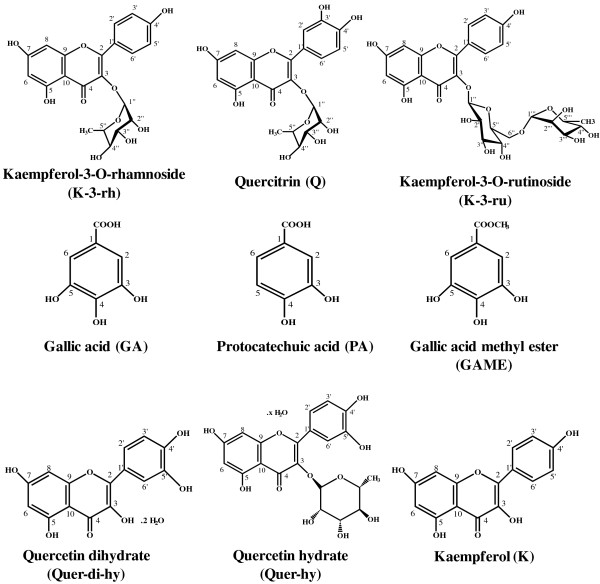
Chemical structures of flavonoids: structures of K-3-rh and other flavonoids are shown (structures not drawn to scale).

**Figure 2:**
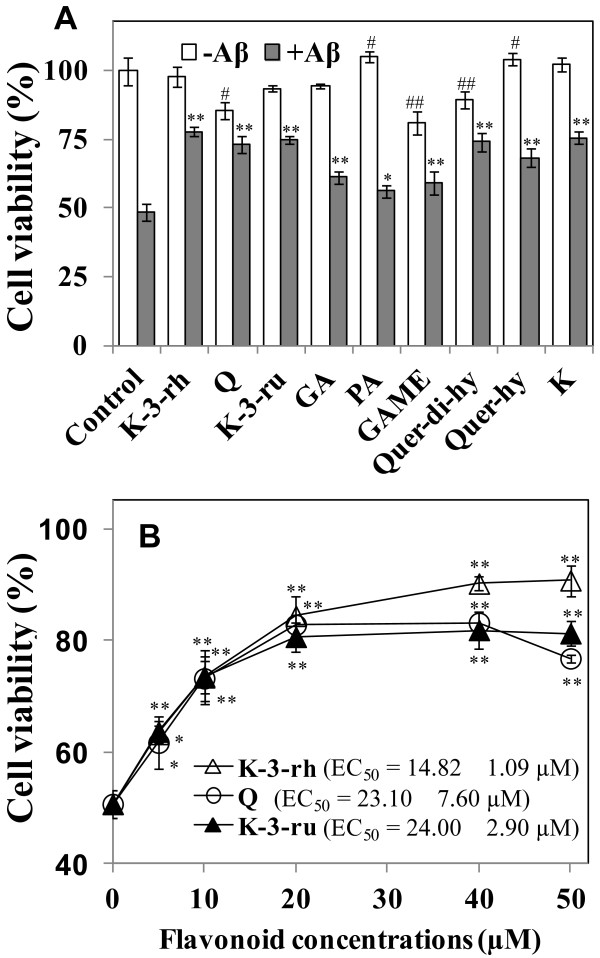
**Effects of flavonoids on Aβ42 induced cytotoxicity. A.** Cells were treated with monomeric Aβ42 (20 μM) for 12 h in the absence or presence of 20 μM of kaempferol-3-O-rhamnoside (K-3-rh), quercitrin (Q), kaempferol-3-O-rutinoside (K-3-ru), gallic acid (GA), protocatechuic acid (PA), gallic acid methyl ester(GAME), quercetin dihydrate(Quer-di-hy), quercetin hydrate (Quer-hy) and kaempferol (K). **B.** Cells were exposed to monomeric Aβ42 (20 μM) in the presence of 0–50 μM of indicated flavonoids for 12 h. Cell viability was assessed using the MTT reduction assay. Error bars indicate the standard deviation of triplicate independent experiments. Significantly different from control without Aβ42 and with Aβ42 (0 flavonoid concentration) groups indicated as #p < 0.05 or ##p < 0.01 and *p < 0.05 or **p < 0.01, respectively.

Flavonoids are suggested to be neuroprotective against several stress or toxic compounds. In the current study, first we screened nine available phenolic compounds (Figure 1) for their protective effects against Aβ42 toxicity to human neuroblastoma SH-SY5Y cells. Treatment with 20 μM of the peptide for 12 h resulted in a decrease of cell viability to ~50%. The most of the flavonoids (20 μM) enhanced cell viability to different extents (Figure 2A). Cell viability enhancement of approximately 25–30% was observed for K-3-rh, Q, K-3-ru, Quer-di-hy, and K. Treatment with compounds such as Q, GAME, and Quer-di-hy alone was found to result in decreased cell viability (Figure 2A). Notably, the most of the polyphenolic flavonoids (Figure 1) found to be more effective than monophenolic compounds for protection of cells against Aβ toxicity (Figure 2A).

Cytoprotective effects of the flavonoids, which showed better efficiency in inhibition of Aβ induced cell death (Figure 2A) and are relatively similar structurally (Figure 1), were further explored for their dose-dependent consequence (Figure 2B). Cell survival was found to be enhanced by increasing the concentration of K-3-rh, where 20 μM of the compound enhanced 30% cell survival (Figure 2B). On the other hand, dose-dependent effects on protection of cells against Aβ toxicity were found to be flattened at > 20 μM for Q and K-3-ru (Figure 2B). This might be due to the toxic effect of Q and K-3-ru on cells, because both of these chemicals were found to decrease cell survival at a higher concentration when applied without Aβ (data not shown). On the other hand, no toxic effect of treatment with K-3-rh alone was observed, even at 50 μM (data not shown). The EC_50_ values calculated as 14.82 ± 1.09, 23.10 ± 7.60 and 24.00 ± 2.90 μM for K-3-rh, Q and K-3-ru, respectively. The results indicated that K-3-rh is an efficient compound for protection against Aβ induced cell death.

### K-3-rh inhibits fibrilogenesis and secondary structural transformation of Aβ42

**Figure 3:**
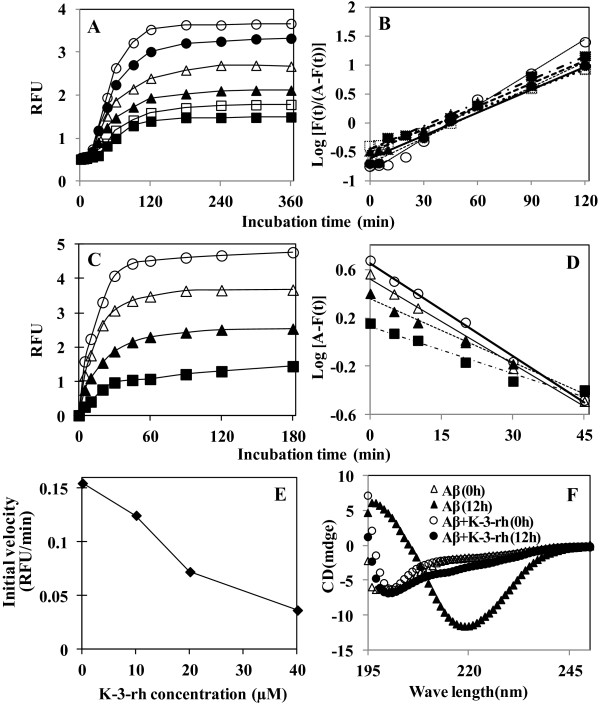
**K-3-rh inhibits polymerization, fibril extension and secondary structural transformation of Aβ42. A.** Inhibition of Aβ42 polymerization. Aβ42 (20 μM) was incubated in PBS at 37°C in the presence of 0 (open circle), 5 (*closed circle*), 10 (open triangle), 20 (*closed triangle*), 40 (open square) and 50 (*closed square*) μM K-3-rh for 0–6 h. IC_50_ was calculated as 30 μM. **B.** Logarithmic plot of *F*(*t*)*/A−F*(*t*) versus reaction time obtained from polymerization assay. **C.** Inhibition of Aβ42 fibril extension. Fresh Aβ42 (20 μM) was added to 1.1 μM preformed seed and incubated at 37°C in the presence of 0 (open circle), 10 (open triangle), 20 (*closed triangle*), and 40 (*closed square*) μM K-3-rh. IC_50_ value was calculated as 20 μM. **D.** Logarithmic plot of *A−F*(*t*) versus reaction time obtained from the fibril extension assay. **E.** Effect of Aβ42 concentrations on the initial rate of Aβ42 fibril extension. **F.** Inhibition of β-sheet transformation of Aβ42. The peptide (20 μM) alone or in the presence of K-3-rh (40 μM) was incubated in PBS at 37°C for 0 or 12 h as indicated. Spectra were obtained by subtracting buffer background as described in Materials and Methods section.

To examine the effects of K-3-rh on fibril formation of Aβ42, 20 μM of peptide was incubated in the presence of several concentrations of K-3-rh and Th-T assay was performed for measurement of fibrillogenesis activity. The fluorescence profiles of Aβ42 aggregation with increasing K-3-rh concentrations are shown in Figure 3A. Aβ42 alone showed a characteristic sigmoidal curve when incubated at 37°C for 6 h [44]. In the presence of 0–50 μM K-3-rh, the final fluorescence level, indicative of the amount of mature fibrils formed, showed a dose-dependent decrease (Figure 3A). Semilogarithmic plots of thioflavin T fluorescence versus time gave a linear relationship for the indicated time (Figure 3B). From these plots, *t/2* values were obtained (see Materials and Methods), which gives us information about the kinetics of the nucleation step during the early stage of fibrillogenesis [40]. It is expected that the *t/2* value would increase if the flavonoids inhibited the nucleation process, the rate-determining step of polymerization. t/2 values for Aβ42 (20 μM) were calculated as 43.07, 45.76, 40.70, 39.22, 39.55 and 34.10 min. in the presence of 0, 5, 10, 20, 40 and 50 μM of K-3-rh, respectively. The small changes of t/2 values in the presence of K-3-rh demonstrated that the marginal effect of the flavonoid in the nucleation step [40]. We also noticed the disappearance of linearity at higher concentrations of flavonoids or upon longer incubation times (Figure 3B), implying the involvement of unidentified processes and, thus, rendering the logistic equation invalid.

The fibril extension experiment was performed by incubating fresh Aβ42 (20 μM) with 1.1 μM preformed Aβ42 fibrils (seed), which showed an increase of fluorescence hyperbolically without lag phase (Figure 3C) [40,44]. The final equilibrium of the curve showed a dose-dependent decrease in the presence of K-3-rh, indicating that the final amount of fibrils formed is decreased in the presence of the flavonoid in a concentration-dependent manner. Four perfect linear logarithmical plots with deferent y intercept were obtained by plotting logarithmic plots of fluorescence difference against times (Figure 3D). The initial rates of Aβ fibril extension, the slopes of the linear portion of the fluorescence profiles, showed a dose-dependent decrease when co-incubated with K-3-rh (Figure 3E). The IC_50_ value against fibril extension was found to be ~20 μM.

Structural transition to form a β-sheet structure is a characteristic feature of Aβ fibrillogenesis [41]. To examine the effect of K-3-rh on secondary structural transformation, Aβ42 (20 μM) was incubated with or without K-3-rh (40 μM) and the presence of β-sheet was detected by CD spectroscopy. CD spectra of Aβ42 alone or with K-3-rh at 0 h, showed a negative absolute value at approximately ~200 nm, suggesting the presence of a largely unfolded peptide at monomeric state with a significant level of random coil (Figure 3F). Upon incubation for 12 h at 37°C, the peptide alone exhibited the shift of a strong signal to negative ellipticity of ~220 nm, indicating the formation of β-sheet structures of the peptide [39]. On the other hand, the transition of random coil to β-sheet structure was not observed in the presence of 20 μM K-3-rh after an incubation period of 12 h (Figure 3F). The results suggest that K-3-rh inhibits β-sheet formation of the peptide. All of these data suggest that K-3-rh did not have a significant impact on fibril nucleation during the lag phase (Figure 3A), however, once the rapid Aβ elongation phase was initiated, the polyphenol inhibited Aβ fibrillogenesis efficiently (Figure 3C and 3E), and it obstructed the secondary structural transition of the peptide to form β-sheet structures.

### K-3-rh causes accumulation of smaller Aβ42 aggregates

**Figure 4:**
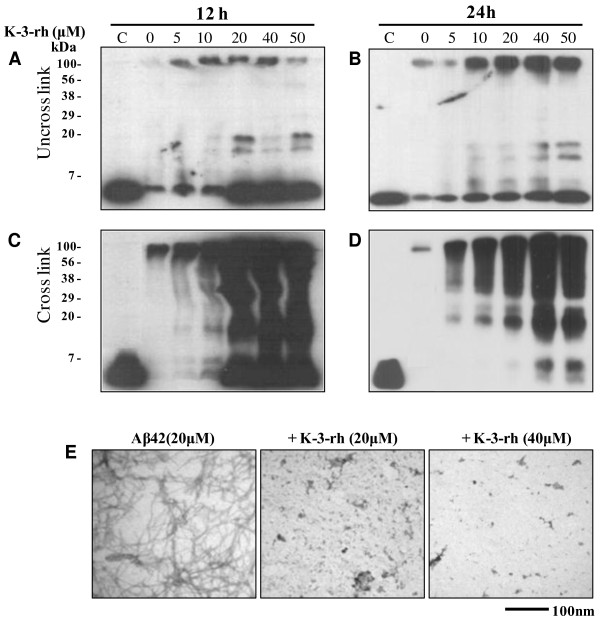
**Accumulation of Aβ42 aggregates in the presence of K-3-rh.** Fresh Aβ42 (20 μM) was incubated in PBS at 37°C for 12 h (**A** and **C**) or 24 h (**B** and **D**), either alone or in the presence of the indicated concentration of K-3-rh. After the incubations, the peptide in the reactions was left uncrosslinked (**A** - **B**) or crosslinked (**C** - **D**) with 0.01% glutaraldehyde before being subjected to 16% SDS-PAGE and the following immunoblotting with anti-Aβ antibody 6E10. Two μl of reaction mixture was loaded for SDS-PAGE. C indicates a fresh Aβ42 (no incubation) as control. The numbers on the left indicate the relative molecular weights of protein markers. **E.** TEM study of the effects of K-3-rh on Aβ42 fibrillogenesis. Twenty μM fresh Aβ42 either alone (left) or in the presence of 20 μM (middle) or 40 μM (right) of K-3-rh were incubated in PBS at 37°C for 12 h. Aβ42 morphology was then visualized by TEM. Scale bar is shown at the bottom.

Polymerization, fibril extension and structural transition studies have shown that K-3-rh inhibits formation of Aβ42 fibrils. The consequences of these inhibitions were characterized by western blot analysis and TEM. In the immunoblot assay, we used Aβ-specific antibody 6E10. A chemical cross-linker was also used to cross-link the Aβ species (Figure 4C-D) prior to SDS-PAGE analysis in order to avoid SDS-induced dissociation of oligomers [39], and compared with non-crosslinking groups (Figure 4A-B).

Incubation with Aβ42 alone for 12 h resulted in the disappearance of almost all monomeric species (Figure 4A, at the bottom of the gel). In the presence of different concentrations of K-3-rh, monomeric predominant bands were observed at the bottom of the gel along with bands around the 20 kDa area of the gel (Figure 4A and 4C), probably the intermediate aggregates of the fibrilogenesis process. These intermediate species, straddling a wide range of sizes, showed a significant increase at higher concentrations (20 μM or more) of flavonoid. In addition, the larger aggregates were found to be enriched when incubation time was increased (Figure 4B), which was clearly observed in crosslinked samples (Figure 4D). Disappearance of monomeric Aβ42 and enrichment of aggregates of several sizes in the presence of K-3-rh were obviously noted upon increasing the incubation times (Figure 4B and 4D). This result suggests that accumulation of several structural species of Aβ occured in the presence of K-3-rh. The data are in good accordance with the earlier reports where accumulations of Aβ oligomers have been observed in the presence of polyphenolic inhibitors [39,45].

The above results indicate that K-3-rh causes accumulation of several intermediates including larger aggregates to small oligomeric species. To confirm the findings, the incubated samples were imaged using TEM in order to visualize the accumulated species. Aβ42 (20 μM) incubated alone at 37°C for 12 h, predominantly composed of typical fibrils (Figure 4E, left panel). In the presence of K-3-rh, however, fibrilar structure was absent, instead, small spherical shaped and some branched structures were observed (Figure 4E, middle and right panels). The morphology and size of these structures are consistent with those of intermediate species previously reported [19,46,47]. The data clearly support the presence of different oligomeric species in Aβ samples incubated with K-3-rh.

### K-3-rh modulated Aβ oligomers are soluble, non-toxic off-pathway aggregates

The above results indicated that K-3-rh inhibits fibrillogenesis and β-sheet formation of Aβ42 peptide. Suppression of Aβ induced cell death in the presence of K-3-rh might be due to these inhibitory processes. On the other hand, inhibitory effects of K-3-rh also modulated several oligomeric intermediates of the peptide (See Figure 4A-E), and the soluble oligomeric Aβ are reported to be more toxic than fresh or fibrilar Aβ [48,49]. Hence, we characterized the K-3-rh accumulated oligomers in order to define their size, structure and cytotoxic properties. First, we obtained the supernatant by centrifuging the Aβ sample preincubated with K-3-rh (40 μM) for 12 h at 37°C. We termed this soluble fraction of Aβ42 as “K-3-rh accumulated oligomers (K-3-rh oligo)”, which were then analyzed and compared with the preformed toxic oligomers [39].

**Figure 5:**
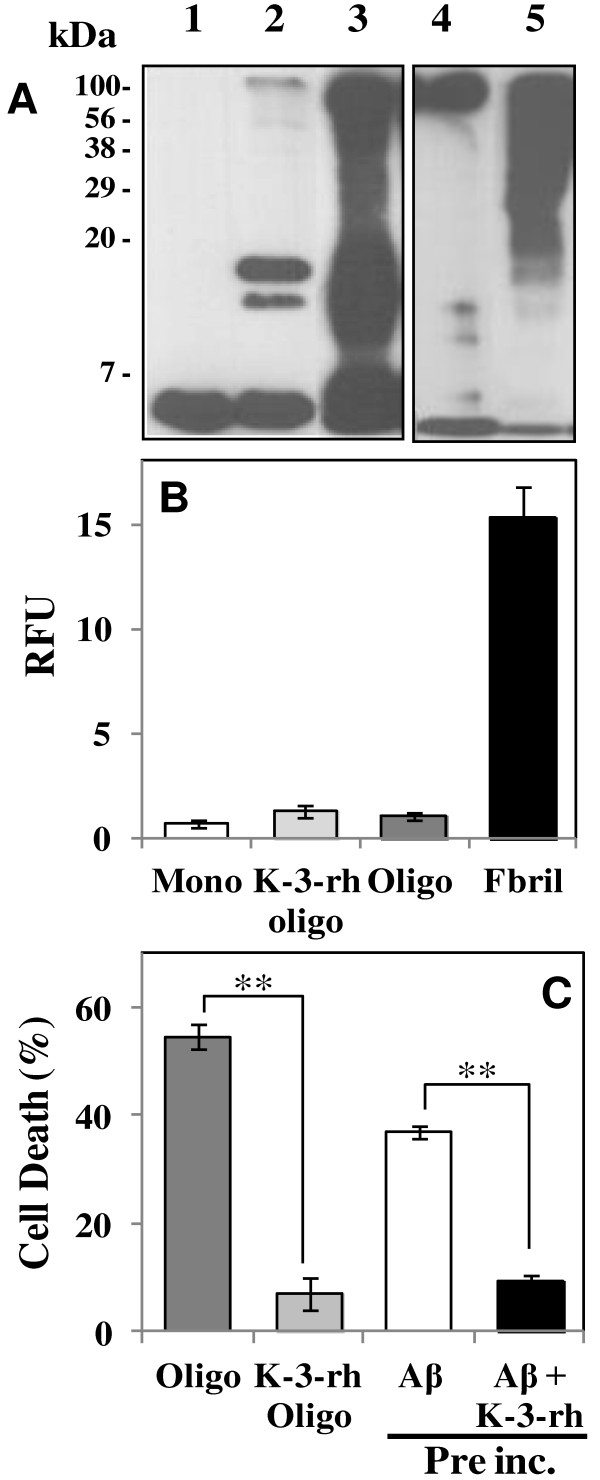
**Biochemical characterization of accumulated Aβ42 species. A.** Immunoblot analysis of Aβ42 oligomers probed with the 6E10 monoclonal antibody: lane 1, fresh Aβ42 as a control; lane 2, K-3-rh accumulated Aβ42 oligomers, obtained in soluble fraction by centrifuging Aβ42(20 μM) and K-3-rh(40 μM) preincubated(12 h) sample; lane 3, Aβ42 of lane 2 cross-linked before the immunoblot assay; lane 4, Aβ42 subjected to the oligomerization process and recovered in the soluble fraction after centrifugation of the mixture(Aβ42 preformed oligomers); lane 5, Aβ42 of lane 4 cross-linked before the immunoblot assay. **B.** Thioflavin T assay of fresh monomeric Aβ42 as control (Mono - white bar), K-3-rh accumulated Aβ42 oligomers (K-3-rh Oligo - light gray bar), Aβ42 preformed oligomers (Oligo - dark gray bar) and Aβ42 mature fibrils (Fibril - black bar). **C.** Decrease of the viability of SH-SY5Y cells (referred to as% of cell death) by Aβ42 preformed oligomers (Oligo - dark gray bar), K-3-rh accumulated Aβ42 oligomers (K-3-rh Oligo - light gray bar) and 20 μM Aβ42 pre-incubated in the absence (Aβ - white bar) or in the presence of 40 μM K-3-rh (Aβ + K-3-rh - black bar) for 12 h. Error bars indicate the standard deviation of triplicate independent experiments and ** indicate significant different between the groups at *p* < 0.01.

K-3-rh accumulated soluble Aβ42 was enriched with mostly smaller aggregates, which was observed as the predominant bands around the <7 kDa and <20 kDa areas of the gel (Figure 5A, lanes 2 and 3). On the other hand, mostly larger species with a size range of ~20 kDa to >100 kDa were visualized in preformed oligomeric Aβ42 (Figure 5A, lane 4 and 5). Both oligomers were Th-T negative (Figure 5B), indicating the absence of β-sheet fibrils among them. While, the inductions of cell death by the oligomers were incomparable (Figure 5C), where preformed oligomers killed ~55% of cells and K-3-rh accumulated oligomers induced only ~7% cell death, suggesting the non-toxic properties of K-3-rh accumulated oligomers. It was noted that, a very small amount of Aβ was found as precipitate during the preparation of a soluble fraction of K-3-rh pre-incubated sample, which may decrease the Aβ concentration in the supernatant. To avoid the variation of Aβ42 concentration and effects of precipitate Aβ42, the preincubated whole samples were also included for measurement of cell death, which also showed a comparable result (~9% cell death by Aβ42 preincubated in the presence of K-3-rh for 12 h) with supernatant sample (Figure 5C). All of these data indicate that K-3-rh modulates the Aβ42 to soluble non-toxic smaller aggregates. The results are in good accordance with our previous report that the polyphenolic flavonoids cause the accumulation of off pathway oligomers [39].

### K-3-rh remodels soluble toxic Aβ oligomers and pre-formed fibrils to non-toxic smaller aggregates

Our results described above suggest that K-3-rh effectively obstructs the Aβ aggregation pathway and modulate to off pathway non-toxic aggregates. Next, we attempt to determine whether the flavonoid is effective for remodeling of the toxic soluble oligomers and mature fibrils. We used preformed soluble oligomers and fibrils as described in the Materials and Methods section, and characterized their aggregation and cytotoxic properties in the absence or presence of K-3-rh.

**Figure 6:**
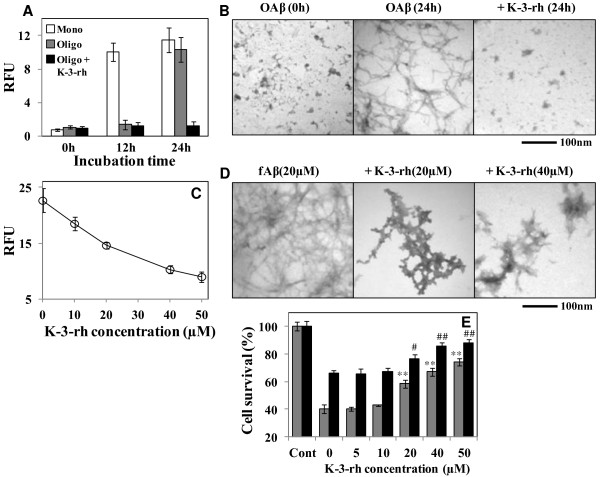
**Preformed oligomers and fibril remodeling activity of K-3-rh. A.** Th-T assay of fresh monomeric Aβ42 as control (white bar), Aβ42 preformed oligomers (gray bar) and Aβ42 preformed oligomers in the presence of 40 μM K-3-rh (black bar) incubated for 0, 12 and 24 h. **B.** TEM study of maturation of Aβ42 preformed oligomers (OAβ) in the absence (left and middle panel) or presence of 40 μM of K-3-rh (right panel). **C.** Dissolution of preformed Aβ42 fibrils in the presence of different concentrations of K-3-rh. The mature Aβ42 fibrils (20 μM) were incubated in the absence or presence of 0, 10, 20, 40 and 50 μM of K-3-rh for 6 h. IC_50_ for fibrils disintegration was found to be > 40 μM. Error bars indicate the standard deviation of triplicate independent experiments. **D.** TEM study of fibril disaggregation properties of K-3-rh. Twenty μM fibrillar Aβ42 (fAβ) was incubated in the absence or presence of 20 and 40 μM of K-3-rh for 12 h and TEM images were obtained. Scale bar is shown at the bottom. **E.** Cells were treated with 20 μM of oligomeric (gray bar) or fibrilar (black bar) Aβ42 for 12 h in the absence or presence of 0–50 μM of K-3-rh. Cell viability was assessed by the MTT reduction assay. Error bars indicate the standard deviation of triplicate independent experiments. Significantly different from only Aβ42 or 0 flavonoid concentration group indicated as **p < 0.01 for oligomers Aβ and ## *p* < 0.01 for fibrilar Aβ.

The preformed oligomers were Th-T negative and stable for longer than 12 h, which converted to Th-T positive fibrillar structure when incubated for 24 h at 37°C (Figure 6A, gray bar and 6B, left and middle panel). In the presence of 40 μM K-3-rh, the oligomers were found to be Th-T negative (Figure 6A, black bar) and had mostly smaller globular structure (Figure 6B, right panel). Similarly, K-3-rh was also found to disaggregate the mature fibrils in a dose-dependent manner when co-incubated with K-3-rh for 12 h (Figure 6C). The IC_50_ value for the disestablishing properties of these fibrils was calculated as >40 μM. The disaggregation of preformed fibrils was also investigated by employing TEM. fAβ42 (20 μM), prepared by incubation at 37°C for three days showed predominantly fibrils (Figure 6D, left panel). The fibrils were converted to a smaller aggregates like shape when incubated in the presence of K-3-rh for 12 h, where dose dependent effects were also observed (Figure 6D, middle and right panels). This finding indicated that K-3-rh disintegrates fibrils and greatly alters the fibrilar shaped to smaller aggregates. These data suggest that K-3-rh remodels the soluble oligomers and preformed fibrils to smaller aggregates. The cytotoxic properties of these converted aggregates were also measured by MTT assay. As shown in Figure 6E, higher concentrations of K-3-rh cause an effective decreased of MTT reduction, where 40 μM of the compound was found to enhance cell survival by ~20% against both preformed oligomers and mature fibrils. All of these data are in good agreement with the previous reports showing that polyphenolic flavonoids remodel soluble oligomers and fibrils to less-toxic smaller aggregates [50,51].

## Discussion

Flavonoids have been widely reported as neuroprotective agents in several diseases including Alzheimer’s and Parkinson’s diseases. The cytoprotective effects of flavonoids can be revealed in three levels: antioxidation, anti-Aβ aggregation, and anti-Aβ induced cell death [52]. Kaempferol and its related flavonoids have been found to be effective against Aβ aggregation [44], and oxidative stress [52]. Yet, the correlation of anti-amyloidogenic activity and anti-cytotoxic effect of these flavonoids remains unclear. In the current study, several flavonoids were screened for their cytoprotective effects against Aβ42 peptide and the mechanisms of anti-amyloidogenic effects were explored using a polyphenolic Keamferol related flavone, K-3-rh. Most of the polyphenolic flavonoids were found to reduce the cytotoxic effects of Aβ to different extents (Figure 2A and 2B). Here, we focused on the properties of Aβ42 aggregation and its relation to cytotoxicity in the presence of K-3-rh in order to explore the mechanisms of the cytoprotective effects of the flavonoid, as well as to better understand the aggregation mechanism of the peptide.

Our findings showed that K-3-rh abrogates cytotoxic effects of Aβ by hindering its on pathway self-assembly. In the presence of K-3-rh, the fibrillogenesis and structural transformation of Aβ peptides were inhibited (Figure 3A-F), which leads to accumulation of several intermediate species (Figure 4A-E). The accumulated species were soluble and relatively smaller in size than toxic oligomers (Figure 5A), devoid of β-sheet fibrillar form (Figures 3F and 5B) and less toxic (Figure 5C), indicating that they might not be the “on pathway” aggregates. The findings are in good agreement with those of previous studies, where polyphenol (−)-epigallocatechin gallate and taiwniaflavone were found to be effective in inhibition of fibrillogenesis of Aβ by directly binding to the unfolded polypeptides and promoted formation of unstructured and nontoxic, off pathway, stable oligomeric structures [39,45]. The K-3-rh and other related flavonoids used in the current study probably redirected the Aβ assembly in similar way to form non toxic off-pathway intermediated structure. The modulation of these aggregates by interaction of K-3-rh, however, might not be specific to unfolded peptides. Our results showed that K-3-rh inhibits fibrillogenesis of Aβ42 peptide (Figure 3A-E), suggesting that the compound might interact with several species during aggregation. The effective inhibition of Aβ42 fibril extension (Figure 3C and 3D) demonstrates the possibility of binding of K-3-rh to the existing fibrils. The consequence of this inhibition is the possible alteration of the aggregation pathway of Aβ by preventing it from misfolding into the β-sheet conformation (Figure 3F). Aromatic interaction between K-3-rh and aromatic residues of the Aβ sequence might be a possible explanation for this inhibitory mechanism. It has been suggested that inhibitory aromatic compounds compete with polypeptide monomers for interaction with growing fibrils, and the irreversible or improved interaction by the inhibitor results in an efficient halt of the fibrillization process [53]. Only the phenolic ring by itself, however, does not efficiently inhibit amyloid formation [54] and further structural elements are necessary for the specific interaction with the amyloidogenic β-sheet conformation for creation of hydrogen bonds that enhance the stability of the inhibitor–protein complex [55]. Structural composition of K-3-rh includes three phenolic groups linked with a rhamnose moiety (Figure 1). The OH groups and rhamnose structure of K-3-rh probably play a supporting role for efficient interaction with β-sheet fibrils of Aβ42 and hence abrogates its further assembly by forming a stable K-3-rh –Aβ42 complex.

The maturation of soluble oligomers is an important event in the Aβ fibrillogenesis pathway. A slight change of agitation condition during Aβ aggregation can result in development of these oligomeric forms to multiple conformers with different biochemical properties rather than following a single nucleation pathway [50]. Small molecules have also been found to convert these soluble oligomers into significantly different structural conformations [56]. Similar remodeling of soluble oligomers by K-3-rh into a less toxic smaller Aβ42 conformer was also noted in the current study (Figure 6A, 6B and 6E). In addition, K-3-rh was also found to disintegrate mature fibrils (Figure 6C) with an IC_50_ value of 40 μM, and to remodel them into smaller aggregates (Figure 6D) with less-toxic properties (Figure 6E). From these results, it seems clear that binding of K-3-rh is not limited to a specific Aβ conformer. However, the result showing that the IC_50_ value of fibril disintegration was higher than that of fibril extension, suggesting the binding preference of K-3-rh to non-fibrillar Aβ conformers. Because both Aβ monomers and soluble oligomers possess similar secondary structure, which is different from β-sheet fibrillar structure, modulation of monomeric Aβ and remodeling of toxic soluble oligomers is expected to occur in a similar manner. In the current study, both monomeric Aβ (Figure 4A-E) and soluble oligomeric Aβ (Figure 6B) were found to convert into smaller aggregates that were less toxic (Figures 5C and 6E). In addition, effective disaggregation properties of polyphenolic glycosidic compounds suggest that glycoside structure is an important determinant for conversion of low molecular weight aggregates from prefibrillar oligomers [51,56,57]. In the current study, the rhamnose moiety of K-3-rh structure may play a partial role in alteration of monomers to low molecular weight soluble non-toxic oligomers (Figures 4A-G and 5A) and preformed oligomers to smaller aggregates (Figure 6B), while other factors may be involved in the process. As another mechanistic approach based on structural similarities between various highly efficient polyphenol inhibitors, and relative to the well-known amyloidogenic dye Congo red, it has been suggested that common efficient polyphenol inhibitors are composed of at least two phenolic rings with two to six atom linkers, and a minimum number of three OH groups on the aromatic rings [55]. These structural similarities imply three-dimensional conformations that are essential for the non-covalent interaction with β-sheet structures [55]. Hence, the non-covalent interaction between Aβ and K-3-rh might be a good speculation for the fibrils remodeling properties of this flavonoid.

## Conclusion

Identification of small molecule inhibitors for inhibition of Aβ aggregation and cytotoxicity is an encouraging approach toward the therapeutic development for treatment of AD. In the current study, we demonstrated that a mono flavonoid furnished with three OH, three phenolic groups conjugated to a rhamnose moiety is an effective molecule for alteration of the on pathway aggregation of Aβ peptide to form non-toxic off pathway conformers. The results support the model of a specific structural conformation for efficient inhibition of amyloidogenic assembly and interaction with β-sheet structure, as well as effectiveness of polyphenol glycosides in abrogation of the amyloidogenic properties of Aβ42 peptide. We suggest that polyphenolic compounds with K-3-rh like structure might be good candidates for incorporation into the therapeutic strategy for treatment of AD.

## Abbreviations

Aβ: Amyloid-β peptide; AD: Alzheimer’s disease; ADDLs: Aβ-derived diffusible ligands; APP: Amyloid precursor protein; CD: Circular dichroism spectroscopy; fAβ: Fibrilar amyloid-β peptide; GA: Gallic acid; GAME: Gallic acid methyl ester; HFIP: 1,1,1,3,3,3-hexafluoro-2-propanol; IC_50_: Concentration of the compound required to reduce the rate of polymerization, fibril extension of fresh Aβ42, or destabilization of preformed Aβ42 fibrils by 50%; K: Keampferol; K-3-rh: Kaempferol-3-O-rhamnoside; K-3-ru: Kaempferol-3-O-rutinoside; MTT: 3-(4,5-dimethylthiazol-2-yl)-2,5-diphenyltetrazolium bromide; OAβ: Oligomeric amyloid-β peptide; PA: Protocatechuic acid; PBS: Phos phate-buffered saline; PVDF: Polyvinylidene difluoride; Q: Quercitrin; Quer-di-hy: Quercetin dihydrate; Quer-hy: Quercetin hydrate; SDS-PAGE: Sodium dodecyl sulfate-polyacrylamide gel electrophoresis; TEM: Transmission electron microscopy; Th-T: Thioflavin T.

## Competing interests

The authors declare that they have no competing interests.

## Authors’ contributions

MGS performed most of the experiments, analyzed data and prepared the manuscript; AT performed some cell death assay, MS helped to purify Aβ, VSR performed some Aβ aggregation experiments, ERW provided some phenolic compounds, SYS designed some experiments and ISP planned experiments, interpreted data and approved the version to be published. All authors read and approved the final manuscript.
